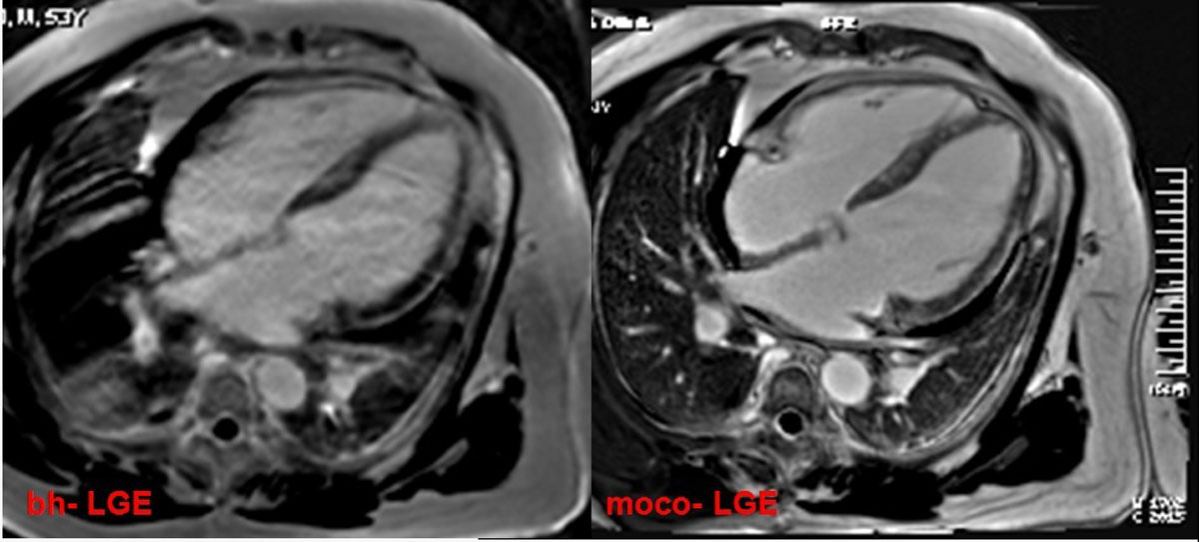# 3.0T motion-corrected single-shot phase sensitive inversion recovery (PSIR) late gadolinium enhancement (LGE) in free-breathing patients compared with conventional segmented breath-held LGE

**DOI:** 10.1186/1532-429X-17-S1-O61

**Published:** 2015-02-03

**Authors:** Lu Lin, Yining Wang, Jian Cao, Lingyan Kong, Jing An, Tianjing Zhang

**Affiliations:** 1 Radiology, Peking Union Medical College Hospital, Beijing, China; 2 Siemens Shenzhen Magnetic Resonance Ltd., Beijing, China

## Background

Novel motion-corrected single-shot phase sensitive inversion recovery (PSIR) late gadolinium enhancement (moco-LGE) cardiovascular MR in 3.0T system may have advantages over conventional segmented breath-held LGE (bh-LGE), especially for vulnerable patients with arrhythmia or respiratory motions.

## Methods

In a consecutive cohort of 58 patients referred for clinical enhanced cardiac MR, bh-LGE and moco-LGE were collected contemporarily with identical image parameters using a 3.0T scanner. The moco-LGE was acquired just after the bh-LGE while the patients were asked to breathe freely. Images were randomized and scored for image quality (1-very poor and not analyzable, 2-poor, 3-acceptable, 4-good, 5-very good) and diagnostic confidence for myocardial LGE (1-low confidence, 2-some confidence, 3-high confidence) separately base on the American Heart Association 17-segmented model. In patients with diagnostic image quality and definite LGE with identifiable margin, the myocardial LGE mass was quantified. Paired t test was used to compare the image quality, diagnostic confidence. Linear regression and correlation plots were used to compare LGE mass.

## Results

55 patients had regular heart rate (HR), the mean HR was 78±14 beats per minute (bpm). The other 3 patient had irregular HR including atrial fibrillation and atrial flutter. In all the patients, the moco-LGE with free-breathing had similarly high image quality (3.9±0.9 vs 3.7±0.9, P=0.410), and diagnostic confidence (2.8±0.3 vs 2.7±0.4, P=0.743) compared with bh-LGE. A total of 16 patients with marked image artifacts in bh-LGE for arrhythmia or respiratory motion, moco-LGE had significantly higher image quality (3.8±0.8 vs 3.0±0.9, P=0.000) and confidence(2.8±0.2 vs 2.4±0.4, P=0.000). The myocardial LGE mass was quantified and compared in 22 patients, the results correlated highly (R^2^=0.95, P=0.000) without bias.

## Conclusions

In general, moco-LGE and bh-LGE have similar image quality and myocardial LGE quantification. In vulnerable patients with marked artifacts of bh-LGE, moco-LGE probably has higher image quality and diagnostic confidence.

## Funding

N/A.

**Figure 1:**